# Molecular Detection and Genotyping of *Enterocytozoon bieneusi* in Beef Cattle in Shanxi Province, North China

**DOI:** 10.3390/ani12212961

**Published:** 2022-10-27

**Authors:** Ya-Ya Liu, Rui-Lin Qin, Jin-Jin Mei, Yang Zou, Zhen-Huan Zhang, Wen-Bin Zheng, Qing Liu, Xing-Quan Zhu, Wen-Wei Gao, Shi-Chen Xie

**Affiliations:** 1 Laboratory of Parasitic Diseases, College of Veterinary Medicine, Shanxi Agricultural University, Taigu 030801, China; 2 State Key Laboratory of Veterinary Etiological Biology, Key Laboratory of Veterinary Parasitology of Gansu Province, Lanzhou Veterinary Research Institute, Chinese Academy of Agricultural Sciences, Lanzhou 730046, China; 3 Research Center for Parasites & Vectors, College of Veterinary Medicine, Hunan Agricultural University, Changsha 410128, China; 4 Key Laboratory of Veterinary Public Health of Higher Education of Yunnan Province, College of Veterinary Medicine, Yunnan Agricultural University, Kunming 650201, China

**Keywords:** *Enterocytozoon bieneusi*, genotypes, prevalence, Shanxi province, beef cattle, zoonosis

## Abstract

**Simple Summary:**

*Enterocytozoon bieneusi* is an important zoonotic intestinal protozoan that can infect humans and many types of animals. Currently, the prevalence and genotyping of *E. bieneusi* in beef cattle in China’s Shanxi province is still unknown. Thus, nested PCR was applied in the present study to investigate the prevalence and genotypes of *E. bieneusi* by examining 401 fecal samples collected from beef cattle in two representative counties of Shanxi province. The results showed that the overall prevalence of *E. bieneusi* in beef cattle in Shanxi province is 22.44% (90/401). Six known genotypes (I, BEB4, J, BEB6, BEB8 and PigSpEb2) and two novel genotypes (designated CSC1 and CSC2) were identified, and genotype I was the predominant genotype in these two counties. Phylogenetic tree analysis indicated that five known genotypes and two novel genotypes were clustered into Group 2, whereas the genotype PigSpEb2 belongs to zoonotic Group 1. To our knowledge, the present study is the first to demonstrate the presence of *E. bieneusi* in beef cattle in Shanxi province, enriching the data on prevalence and genotypes of *E. bieneusi* in beef cattle and providing baseline data for executing intervention measures to control it in the study regions.

**Abstract:**

*Enterocytozoon bieneusi* is an intracellular pathogen that can parasitize humans and a variety of animals. The infection of *E. bieneusi* in most hosts is asymptomatic, but in immunocompromised individuals, it can lead to serious complications such as acute diarrhea, dehydration, and even death. However, no data on the prevalence and genotyping of *E. bieneusi* in beef cattle in Shanxi province are currently available. In this study, a total of 401 fecal samples were collected from beef cattle in farms from two representative counties—Qi county and Jishan county—in Shanxi province, north China. Nested PCR was applied to determine the prevalence and genotypes of *E. bieneusi* by amplifying and sequencing the internal transcribed spacer (ITS) regions of the rRNA gene. A total of 90 out of 401 samples were detected as *E. bieneusi*-positive, with 22.44% overall prevalence of *E. bieneusi* in beef cattle in Shanxi province. The highest prevalence of *E. bieneusi* was detected in calves (28.67%, 41/143) and male beef cattle (28.13%, 54/192). Statistical analysis revealed that the prevalence of *E. bieneusi* was significantly associated with gender and age factors (*p* < 0.05), but without any statistical difference among regions. Moreover, six known *E. bieneusi* genotypes (BEB4, BEB6, BEB8, J, I, and PigSpEb2) and two novel genotypes (designated CSC1 and CSC2) were identified by analysis of ITS sequences, and genotype I was the predominant genotype in these two counties. Phylogenetic analysis showed that five known genotypes and two novel genotypes were clustered into Group 2, but PigSpEb2 belonged to Group 1. To our knowledge, the present study demonstrated the presence and identified genotypes of *E. bieneusi* in beef cattle in Shanxi province for the first time, extending the data on prevalence and genotypes of *E. bieneusi* in beef cattle and providing baseline data for executing intervention measures to control it in the study regions.

## 1. Introduction

Currently, approximately 220 genera and 1700 species of microsporidians have been reported, of which 17 species of microsporidian have been commonly reported in humans [1]. Among these species, *Enterocytozoon bieneusi*, *Encephalitozoon cuniculi*, *Encephalitozoon intestinalis* and *Encephalitozoon hellem* are the four major pathogens responsible for human microsporidiosis [2]. *E. bieneusi* is one of the most diagnosed zoonotic pathogens and responsible for more than 90% of microsporidiosis in humans [2,3,4,5]. As an opportunistic parasite, *E. bieneusi* infection can cause acute diarrhea in immunocompromised individuals, and even death in some AIDS patients [6]. Humans and animals can be infected with *E. bieneusi* by ingestion of spores maintained in water, vegetable and food, via the fecal-oral route. Due to the minute size of *E. bieneusi* spores, nested PCR has been applied to amplify the internal transcribed spacer (ITS) regions of the rRNA gene as a high-efficiency method to investigate molecular epidemiology and genotypes of *E. bieneusi* [7]. Over 500 identified genotypes of *E. bieneusi* were divided into 11 major genetic groups (named as Group 1 to 11). Group 1 and Group 2 include the majority of zoonotic genotypes, and Group 3 to 11 appear to belong to host-adapted groups [7].

In 2000, *E. bieneusi* was detected for the first time in cattle with diarrhea [8]. Since then, a number of studies have reported cases of *E. bieneusi* infection in cattle worldwide [9,10,11,12]. As with other animals, *E. bieneusi* infection in cattle can cause chronic diarrhea and wasting syndrome, especially among calves with incompetent immune systems [13]. Furthermore, past studies have reported *E. bieneusi* in raw milk of cattle [14,15] and in the environment [16], indicating that it may be a public health concern. A recent review indicated that *E. bieneusi* prevalence in cattle is 14% worldwide, with the highest prevalence in calves [17]. To date, approximately 50 genotypes of *E. bieneusi* have been detected in cattle in China, and most of these genotypes belong to Group 1 (e.g., genotype D, IV, Peru6, CHN4 and EbpC) and Group 2 (e.g., genotype J, I, BEB4, BEB6, CHN3 and CS-4) [13,18].

In Shanxi province, north China, the beef cattle industry is developing rapidly due to growing consumer demand for beef. However, little is known about the prevalence and genotypes of *E. bieneusi* in beef cattle in the province. Therefore, this study seeks to determine the prevalence and genotypes of *E. bieneusi* in beef cattle in Shanxi province. 

## 2. Materials and Methods

### 2.1. Study Sites and Sampling

**Figure 1:**
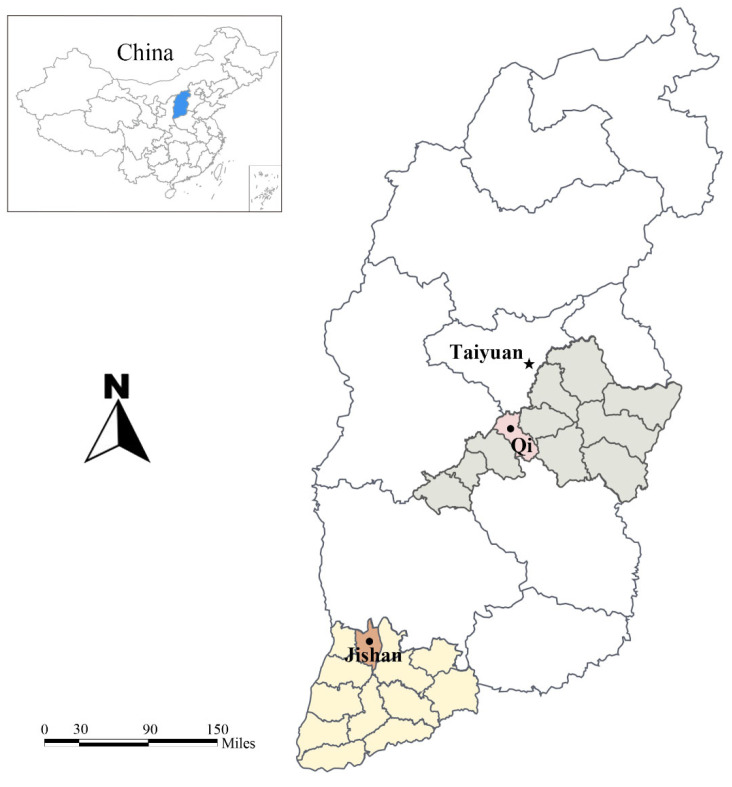
Sampling locations (Qi county and Jishan county) of beef cattle feces in Shanxi province, north China.

In this study, a total of 401 fecal samples were randomly collected from beef cattle without diarrhoea in Qi county (*n* = 177) and Jishan county (*n* = 224) in Shanxi province (Figure 1). Approximately 10 g of each fecal sample was collected from the center of fresh excrement from beef cattle using polyethylene (PE) gloves, then marked with information about the region, age and gender of the animal. All samples were kept in a Styrofoam box with ice packs and transported to the laboratory, where they were stored in a freezer at −20 °C until DNA extraction.

### 2.2. DNA Extraction and PCR Amplification

Approximately 200 mg of each fecal sample was used to extract genomic DNA using the commercial E.Z.N.A.^®^ Stool DNA Kit (Omega, Biotek Inc., Norcross, GA, USA) according to the manufacturer’s protocols. To determine the prevalence and genotypes of *E. bieneusi* in beef cattle, the 389 bp fragment containing the ITS region was amplified by nested PCR using the primers reported in a previous study [19]. The PCR reaction mixture (25 μL) was composed of 15.25 μL of ddH_2_O, 0.2 mM of dNTP, 2.5 μL of 10 × PCR Buffer (Mg^2+^ free), 1.5 mM of MgCl_2_, 1 μL of each primer, 0.125 μL of *Ex*-Taq (5 U/μL), and 1.5 μL of genomic DNA (for the primary PCR) or the primary PCR product (for the secondary PCR). The primary and secondary PCR amplification conditions and cycling parameters were as follows: initial denaturation at 94 °C for 5 min, then followed by 35 cycles of denaturation at 94 °C for 45 s, annealing at 57 °C for 45 s, extension at 72 °C for 1 min, and a final extension at 72 °C for 10 min. The positive control (DNA of the BEB6 genotype from sheep) and negative control (reagent-grade water) were added to each PCR round to ensure the reliability of the results. The secondary PCR products were checked by electrophoresis in 2.5% agarose gel with ethidium bromide.

### 2.3. Sequence Analysis and Phylogenetic Reconstruction

The *E. bieneusi* positive PCR products were sent to Sangon Biotech Co. Ltd. (Shanghai, China) for two-way sequencing. The ITS sequences of *E. bieneusi* were checked first according to the sequencing peak map using Chromas V2.6, and aligned with other relevant sequences in GenBank database using basic local alignment search tool (BLAST) for identification of *E. bieneusi* genotypes. Following the established nomenclature system of *E. bieneusi*, only 243 bp of the ITS sequences was retained for identification of *E. bieneusi* novel genotypes [20]. The phylogenetic relationship of ITS genotypes was established by the Neighbor-joining (NJ) method using the Kimura-2-parameter model in MEGA 7. The bootstrap value was set to 1000 times to evaluate the robustness of clusters [7]. The representative *E. bieneusi* genotypes obtained in this study were deposited in the GenBank database with accession numbers OM101097-OM101104.

### 2.4. Statistical Analysis

In this study, the chi-square test was used to assess the difference in *E. bieneusi* prevalence in region, age and gender groups using software SPSS 26.0 (SPSS Inc., Chicago, IL, USA). Odds ratios (ORs) and their 95% confidence intervals (95%CI) were also explored to estimate the strength of the association between *E. bieneusi* prevalence and the test conditions. It is considered to be statistically significant when the *p*-value is less than 0.05.

## 3. Results

### 3.1. Prevalence of E. bieneusi in Beef Cattle

In this study, 90 out of 401 fecal samples of beef cattle were detected *E. bieneusi*-positive by electrophoresis and sequencing, and the overall prevalence of *E. bieneusi* is 22.44% (90/401) (Table 1). *E. bieneusi* infection was detected in every region, gender and age group that was assessed. The prevalence of *E. bieneusi* in Jishan county (24.55%, 55/224) was higher than that in Qi county (19.77%, 35/177), but the difference was not statistically significant (χ2 = 1.298, df = 1, *p* = 0.255). In gender groups, a statistically significant difference in *E. bieneusi* prevalence (χ2 = 6.830, df = 1, *p* < 0.01) was found between female beef cattle (17.22%, 36/209) and male beef cattle (28.13%, 54/192). The prevalence of *E. bieneusi* in young cattle (≤ 12 months) (28.67%, 41/143) was higher than that in adult cattle (> 12 months) (18.99%, 49/258). This was also statistically significant (χ2 = 4.952, df = 1, *p* < 0.05).

### 3.2. E. bieneusi Genotype Contribution in Beef Cattle

**Figure 2:**
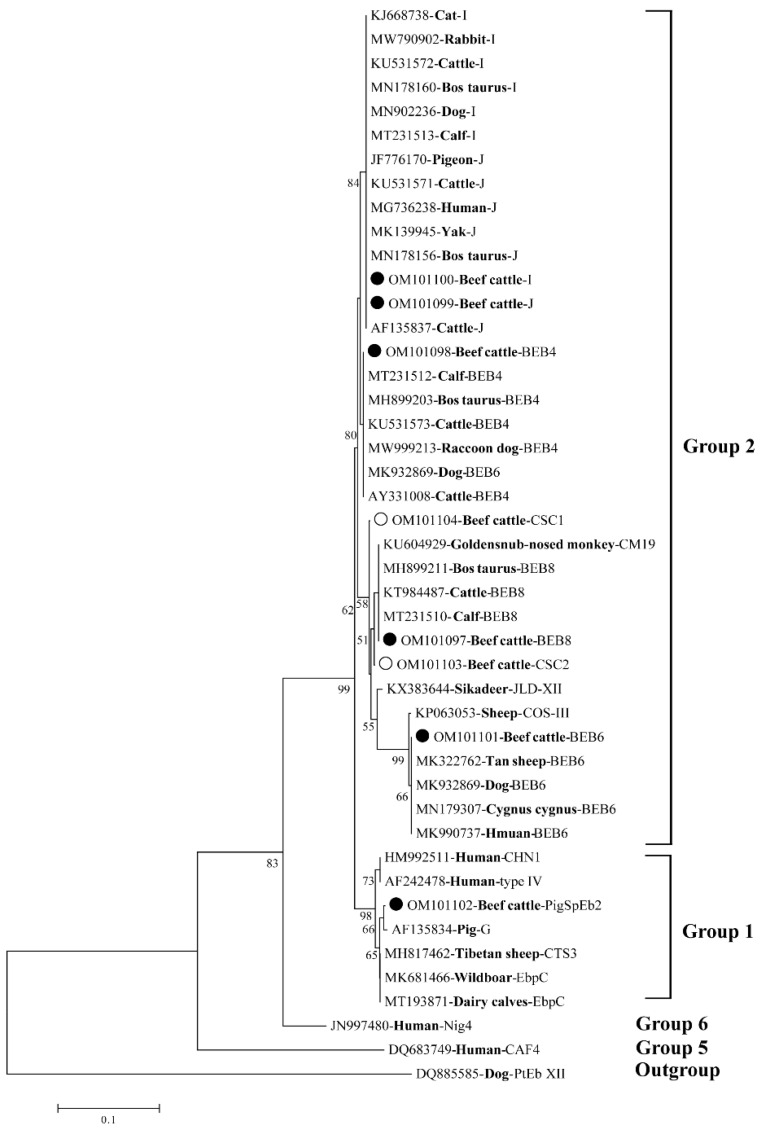
Phylogenetic relationships of *Enterocytozoon bieneusi* genotypes identified in this study and in previous reports. The six known genotypes and two novel genotypes identified in this study are marked with black circles and unfilled circles, respectively. Numbers on branches are the percentage of bootstrap values from 1000 replications.

Among the 90 *E. bieneusi*-positive samples sequenced in the present study, eight distinct *E. bieneusi* genotypes were successfully identified by comparative analysis of ITS sequences (Table 2). Of these genotypes, six were known genotypes (I, J, BEB8, BEB4, BEB6 and PigSpEb2). In addition, two novel genotypes were identified and designated as CSC1 and CSC2. To further explore the pattern of *E. bieneusi* transmission in the two areas under study, the prevalence and genotypes of *E. bieneusi* among different risk factors are listed in Table 2. Among the eight identified genotypes, genotype I was the most prevalent genotype in both areas (55.56%, 50/90), followed by BEB4 (11.43%, 20/90), J (11.11%, 10/90), BEB8 (4.44%, 4/90), BEB6 (3.33%, 3/90), PigSpEb2 (1.11%, 1/90), CSC1 (1.11%, 1/90) and CSC2 (1.11%, 1/90). Moreover, genotype I was identified as the most predominant genotype in male beef cattle (57.41%, 31/54) and adult beef cattle (65.31%, 32/49) (Table 2). Interestingly, all eight genotypes were detected in Qi county, but only three genotypes (I, J and BEB4) were found in Jishan county. As shown in Figure 2, phylogenetic tree analysis indicated that seven genotypes including the two novel genotypes (CSC1 and CSC2) were clustered into Group 2. The exception was genotype PigSpEb2, which belonged to Group 1.

## 4. Discussion

*E. bieneusi* is an important obligate intracellular pathogen that threatens the development and economic status of animal husbandry. The present study revealed an overall *E. bieneusi* prevalence of 22.44% in beef cattle in Shanxi province. As shown in Table 3, the prevalence of *E. bieneusi* in beef cattle detected in this study was higher than that in cattle in most other provinces of China, except for Xinjiang Uygur Autonomous Region (52.00%, 130/250) [21], Shanghai city (26.45%, 214/809) [22], Henan province (24.35%, 214/879) [13], Heilongjiang province (30.08%, 40/133) [23] and Ningxia Autonomous Region (45.87%, 50/109) [13]. We speculate that the difference in *E. bieneusi* prevalence in cattle among different provinces may result from differences in the grazing conditions. Grazing is the major feeding mode in study areas, which increases the chance of cattle ingesting *E. bieneusi* via the environment. Moreover, according to the present study, the prevalence of *E. bieneusi* in beef cattle in Shanxi province was higher than that in most other countries, although lower than that in some regions of the USA (36.17%, 17/47; 34.80%, 285/819; 22.94%, 131/571) [17,24,25].

In the present study, there was no significant difference in the prevalence of *E. bieneusi* in beef cattle between Qi county and Jishan county (*p* = 0.255). However, significant differences in prevalence were found among beef cattle of different genders and ages. In this study, the prevalence of *E. bieneusi* in calves (28.7%) was higher than that in adult cattle (19.0%), a finding consistent with previous studies in Shaanxi province [36], Ningxia Autonomous Region and Henan province [13]. We reasoned that the susceptibility of calves to *E. bieneusi* may be related to their immature immune systems. In this study, higher *E. bieneusi* prevalence was reported in male beef cattle (28.1%) than that in female beef cattle (17.2%). However, the literature regarding differences in the prevalence of *E. bieneusi* in cattle is limited. Only one publication reported a higher prevalence of *E. bieneusi* in female cattle (0.67%, 5/751) than that in male cattle (0%, 0/90), with no statistical difference [39]. Therefore, the underlying reasons for difference in prevalence between genders is still unclear, and the potential role played by gender in *E. bieneusi* transmission in cattle needs to be supported by more data.

To better understand the *E. bieneusi* genotype distribution in the two study areas, six known genotypes (I, BEB4, J, BEB8, BEB6 and PigSpEb2) and two novel genotypes (CSC1 and CSC2) of *E. bieneusi* were identified, of which genotype I was the prevalent genotype across study areas, genders and age groups (Table 2). More genotypes of *E. bieneusi* were detected in Qi county than in Jishan county. However, there was no significant difference in genotype abundance among gender and age groups, except for genotype PigSpEb2 (*n* = 1), CSC1 (*n* = 1), CSC2 (*n* = 1). A recent comprehensive review indicated that genotype I, J, BEB4, BEB6 and BEB8 are the most common genotypes detected in cattle worldwide [7]. Moreover, genotype I has also been identified as the predominant genotype in cattle in Shaanxi province and Tibet Autonomous Region in China [36,37], although genotype J was the most frequently reported genotype in other provinces of China (Table 3). The genotypes J, I, BEB4 and BEB6 are the most frequently detected zoonotic genotypes in both humans and animals, posing a potential public health risk [17,25,40,41,42]. In 2021, genotype PigSpEb2 was first detected in pigs in Spain, and its pathogenicity is still unknown [43]. We speculate that several factors could be responsible for these differences, including husbandry practice, rearing systems, hygiene conditions and cattle breeds.

Compared with reference sequence (MN728943 from ITS genotype BEB6), two novel genotypes CSC1 and CSC2 generated similar sequences with 10 and 11 single nucleotide polymorphisms (SNPs), respectively. Therefore, a phylogenetic tree was constructed to assess the potential zoonotic risk of *E. bieneusi* genotypes. As shown in Figure 2, five of the known genotypes detected—and the two novel genotypes (CSC1 and CSC2)—were all clustered into Group 2. The sixth known genotype detected, PigSpEb2, was the exception. These results revealed that beef cattle can be a potential reservoir host for human infection with *E. bieneusi*. Therefore, appropriate public health interventions should be made in these two study areas. For example, regular detection and sterilization should be applied. Distributing free medication (e.g., albendazole or fumagillin) is also an effective way to reduce the risk of *E. bieneusi* infection in cattle and other animals [44].

## 5. Conclusions

The present study revealed an overall *E. bieneusi* prevalence of 22.44% in beef cattle in Shanxi province, north China, with significant differences across gender and age groups. Six known *E. bieneusi* genotypes (I, BEB4, J, BEB8, BEB6 and PigSpEb2) and two novel genotypes (CSC1 and CSC2) were identified, with genotype I being the most prevalent genotype in the study areas. Phylogenetic analysis suggested that two novel *E. bieneusi* genotypes have the potential to be transmissible to humans and other animals. These findings have important implications for carrying out intervention measures against *E. bieneusi* infection in beef cattle and other animals.

## Figures and Tables

**Table 1:** Factors associated with the prevalence of *Enterocytozoon bieneusi* in beef cattle in this study.

Factor	Categories	No. Tested	No. Positive	Prevalence% (95%CI)	OR (95%CI)	*p*-Value
Area	Qi	177	35	19.77 (13.91–25.64)	1	0.255
	Jishan	224	55	24.55 (18.92–30.19)	1.32 (0.82–2.13)	
Gender	Male	192	54	28.13 (21.77–34.48)	1.88 (1.17–3.03)	<0.01
	Female	209	36	17.22 (12.11–22.34)	1	
Age	Age > 12 months	258	49	18.99 (14.21–23.78)	1	<0.05
	Age ≤ 12 months	143	41	28.67 (21.26–36.08)	1.71 (1.06–2.76)	
Total		401	90	22.44 (18.36–26.53)		

**Table 2:** Genotype distribution of *Enterocytozoon bieneusi* identified in beef cattle in this study.

Factors	Categories	No. Positive/Total	Prevalence (%)	ITS Genotypes (n)
Region	Qi	35/177	19.77	I (15), J (6), BEB8 (4), BEB4(4), BEB6 (3), PigSpEb2 (1), CSC1 (1), CSC2 (1)
	Jishan	55/224	24.55	I (35), BEB4 (16), J (4)
Age	Age ≤ 12 months	41/143	28.67	I (18), BEB4 (11), J (5), BEB8 (3), BEB6 (3), PigSpEb2 (1)
	Age > 12 months	49/258	18.99	I (32), BEB4 (9), J (5), BEB8 (1), CSC1 (1), CSC2 (1)
Gender	Male	54/192	28.13	I (31), BEB4 (14), J (4), BEB8 (2), BEB6 (2), PigSpEb2 (1)
	Female	36/209	17.22	I (19), J (6), BEB4 (6), BEB8 (2), BEB6 (1), CSC1 (1), CSC2 (1)
Total		90/401	22.44	I (50), BEB4 (20), J (10), BEB8 (4), BEB6 (3), PigSpEb2 (1), CSC1 (1), CSC2 (1)

**Table 3:** Prevalence and prevalent genotype of *Enterocytozoon bieneusi* in cattle in China.

Province	Gene	No. Positive/Examined	Prevalence (%)	Prevalent Genotype	Host	References
Anhui	ITS	40/955	4.19	J	Cattle	[26]
Gansu	ITS	4/353	1.13	BEB4	Yak	[27]
Guangdong	ITS	61/388	15.72	J	Pre-weaned cattle	[28]
Heilongjiang	ITS	30/423	7.09	J	Cattle	[29]
Hainan	ITS	31/314	9.87	EbpC	Cattle	[30]
Henan	ITS	33/277	11.91	BEB6	Dairy cattle	[31]
Henan	ITS	214/879	24.35	J	Dairy cattle	[13]
Heilongjiang	ITS	40/133	30.08	O	Dairy cattle	[23]
Jiangxi	ITS	30/556	5.40	D	Cattle	[32]
Jiangsu	ITS	177/1366	12.96	J	Dairy cattle	[33]
Ningxia	ITS	50/109	45.87	J	Dairy cattle	[13]
Qinghai	ITS	23/327	7.03	BEB4	Yak	[34]
Qinghai	ITS	40/544	7.35	J	Yak	[35]
Shanghai	ITS	214/809	26.45	J	Pre-weaned cattle	[22]
Shaanxi	ITS	73/371	19.68	I	Cattle	[36]
Tibet	ITS	11/442	2.49	I	Cattle	[37]
Xinjiang	ITS	85/514	16.54	J	Dairy cattle	[38]
Xinjiang	ITS	130/250	52.00	J	Dairy cattle	[21]
Yunnan	ITS	5/841	0.59	- ^a^	Dairy cattle	[39]

^a^: indicated no data available.

## Data Availability

The data sets supporting the results of this article have been submitted to the GenBank and accession number shown in the article.
